# Effects of rAAV-mediated FGF-2 gene transfer and overexpression upon the chondrogenic differentiation processes in human bone marrow aspirates

**DOI:** 10.1186/s40634-016-0052-6

**Published:** 2016-07-29

**Authors:** Janina Frisch, Jagadeesh K. Venkatesan, Ana Rey-Rico, Adam M. Zawada, Gertrud Schmitt, Henning Madry, Magali Cucchiarini

**Affiliations:** 1 Center of Experimental Orthopaedics, Saarland University Medical Center, Kirrbergerstr. Bldg 37, D-66421 Homburg/Saar, Germany; 2 Department of Internal Medicine IV, Saarland University Medical Center, Homburg/Saar, Germany; 3 Department of Orthopaedic Surgery, Saarland University Medical Center, Homburg/Saar, Germany

**Keywords:** Cartilage repair, Gene therapy, Human bone marrow aspirates, rAAV, FGF-2

## Abstract

**Background:**

Application of genetically modified bone marrow concentrates in articular cartilage lesions is a promising approach to enhance cartilage repair by stimulating the chondrogenic differentiation processes in sites of injury.

**Method:**

In the present study, we examined the potential benefits of transferring the proliferative and pro-chondrogenic basic fibroblast growth factor (FGF-2) to human bone marrow aspirates in vitro using the clinically adapted recombinant adeno-associated virus (rAAV) vectors to monitor the biological and chondrogenic responses over time to the treatment compared with control (*lacZ*) gene application.

**Results:**

Effective, significant FGF-2 gene transfer and expression via rAAV was established in the aspirates relative to the *lacZ* condition (from ~ 97 to 36 pg rhFGF-2/mg total proteins over an extended period of 21 days). Administration of the candidate FGF-2 vector led to prolonged increases in cell proliferation, matrix synthesis, and chondrogenesis but also to hypertrophic and terminal differentiation in the aspirates.

**Conclusion:**

The present evaluation shows the advantages of rAAV-mediated FGF-2 gene transfer to conveniently modify bone marrow concentrates as a future approach to directly treat articular cartilage lesions, provided that expression of the growth factor is tightly regulated to prevent premature hypertrophy in vivo.

## Background

The adult articular cartilage has a limited ability for self-repair (Buckwalter 1998) due to the absence of vascularization in this particular joint tissue that may be a potential source of chondrogenerative cells such as mesenchymal stem cells (MSCs) (Orth et al. 2014). While progenitor cells can be made accessible within sites of injury by procedures like marrow stimulation (Dewan et al. 2014; Madry et al. 2011), the outcomes of such techniques do not match the expected native hyaline cartilage and instead, a fibrocartilaginous repair tissue is formed in the lesions with mostly type-I versus type-II collagen and proteoglycans (Dewan et al. 2014). One-step administration of bone marrow concentrates containing chondrogenically competent MSCs (Anam and Davis 2013; Lennon et al. 2000) within cartilage defects rather than of isolated and/or expanded MSCs raised important attention as a new, convenient option to improve cartilage repair (Orth et al. 2014). Still, even when using such a straightforward approach (Enea et al. 2013; Gigante et al. 2012; Kim et al. 2014; Orozco et al. 2013; Skowronski et al. 2013; Slynarski et al. 2006; Wakitani et al. 2004), an original (structural and functional) articular cartilage repair tissue could not be regenerated in patients, demonstrating the necessity to develop improved therapeutic strategies to manage cartilage injuries.

The modification of bone marrow aspirates by gene transfer technologies is a promising, new avenue of translational research as a means to enhance the chondrogenic responses to injury by overexpressing target candidates with potential reparative activities (Cucchiarini et al. 2014; Frisch et al. 2015b). In this regard, a number of studies revealed the feasibility of applying adenoviral vectors at very high doses (10^10^-10^11^) to promote the expression of reporter genes (*E. coli* β-galactosidase, firefly luciferase, green fluorescent protein) (Pascher et al. 2004) or therapeutic sequences including the transforming growth factor beta (TGF-β) (Ivkovic et al. 2010), bone morphogenetic protein 2 (BMP-2) (Sieker et al. 2015), and Indian hedgehog (IHH) (Sieker et al. 2015) in marrow aspirates from rabbits and sheep but only for relatively short periods of time (some days). Instead, we recently provided evidence that recombinant adeno-associated virus (rAAV) vectors are adapted gene vehicles to competently transduce human marrow concentrates over extended periods of time at very high efficiencies (~90 % for at least 125 days) using much lower vector doses (8 × 10^5^) and without detrimental effect (Rey-Rico et al. 2015) compared with the more immunogenic adenoviral vectors (Frisch et al. 2015b). We showed for instance that effective rAAV-mediated overexpression of the transcription factor SOX9 (Rey-Rico et al. 2015) or of insulin-like growth factor I (IGF-I) (Frisch et al. 2015a) was capable of activating the chondrogenic processes in freshly prepared human marrow samples. Here, we focused on delivering the basic fibroblast growth factor (FGF-2) to human marrow aspirates in light of the proliferative and pro-chondrogenic activities of this agent in isolated human MSCs when applied as a recombinant molecule (Solchaga et al. 2010; Solchaga et al. 2005; Tsutsumi et al. 2001) or upon administration of the current rAAV FGF-2 vector (Cucchiarini et al. 2011). The present study reveals that delivery of rAAV allows for an effective and durable production of FGF-2 in human marrow concentrates, allowing for enhanced levels of cell proliferation, matrix synthesis, and chondrogenic differentiation in such samples. Processes of hypertrophic and terminal differentiation were also activated, suggesting that a critical regulation of transgene expression will be needed for FGF-2 using rAAV vectors prior to translation of the approach in vivo. Nevertheless, these findings reflect the value of rAAV gene transfer to modify marrow concentrates for future procedures of implantation in sites of cartilage damage.

## Methods

### Reagents

All reagents were from Sigma (Munich, Germany), unless otherwise identified. Recombinant TGF-β (rTGF-β) was purchased at Peprotech (Hamburg, Germany) and the dimethylmethylene blue dye at Serva (Heidelberg, Germany). The anti-FGF-2 (C-18) and anti-SOX9 (C-20) antibodies were from Santa Cruz Biotechnology (Heidelberg, Germany), the anti-type-II collagen (II-II6B3) antibody from the NIH Hybridoma Bank (University of Iowa, Ames, USA), the anti-type-I collagen (AF-5610) antibody from Acris (Hiddenhausen, Germany), and the anti-type-X collagen (COL-10) antibody from Sigma. Biotinylated secondary antibodies and the ABC reagent were obtained from Vector Laboratories (Alexis Deutschland GmbH, Grünberg, Germany). The FGF-2 Quantikine ELISA (DFB50) was from R&D Systems (Wiesbaden, Germany) and the type-II, −I, and -X collagen ELISAs from Antibodies-Online (Aachen, Germany).

### Plasmids and rAAV vectors

All plasmids are based on the same parental AAV-2 genomic clone, pSSV9 (Samulski et al. 1987; Samulski et al. 1989). rAAV-*lacZ* carries the *lacZ* gene encoding the *E. coli* β-galactosidase (β-gal) and rAAV-hFGF-2 a human basic fibroblast growth factor (hFGF-2) cDNA fragment (480 bp), both under the control of the cytomegalovirus immediate-early (CMV-IE) promoter (Cucchiarini et al. 2011; Frisch et al. 2015a; Rey-Rico et al. 2015). Conventional (not self-complementary) rAAV vectors were packaged using the 293 adenovirus-transformed embryonic kidney cell line. Helper functions were provided by Adenovirus 5 in combination with rep and cap functions of a pAd8 helper plasmid as previously described (Cucchiarini et al. 2011; Frisch et al. 2015b). Purification, dialysis, and titration of the vector preparations via real-time PCR were performed, averaging 10^10^ transgene copies/ml with approximately 1/500 functional recombinant viral particles (Cucchiarini et al. 2011; Frisch et al. 2015b; Rey-Rico et al. 2015).

### rAAV-mediated gene transfer

Bone marrow was aspirated from the distal femurs of patients undergoing total knee arthroplasty (~10 ml, n = 3). Aspirates were immediately aliquoted in a volume of 100 μl per well in 96-well plates and transduced with 40 μl vector (i.e. 8 × 10^5^ functional recombinant viral particles, MOI = 10 ± 3) (Frisch et al. 2015b; Rey-Rico et al. 2015). Samples were incubated for up to 21 days with chondrogenic medium (4.5 g/l DMEM high glucose, 100 U/ml penicillin, 100 μl/ml streptomycin, 6.25 μg/ml insulin, 6.25 μg/ml transferrin, 6.25 μg/ml selenious acid, 5.35 μg/ml linoleic acid, 1.25 μg/ml BSA, 1 mM sodium pyruvate, 37.5 μg/ml ascorbate 2-phosphate, 10^−7^ M dexamethasone, and 10 ng/ml TGF-β3) with careful weekly medium change as previously described (Cucchiarini et al. 2011; Frisch et al. 2015b).

### Transgene expression

FGF-2 production was monitored by ELISA at the denoted time points by absorbance measurements on a GENios spectrophotometer/fluorometer (Tecan, Crailsheim, Germany) and by immunohistochemistry using a specific FGF-2 antibody, a biotinylated secondary antibody, and diaminobenzidine as a chromogen (ABC method) (Cucchiarini et al. 2011). A control condition with omission of the primary antibody was included to check for secondary immunoglobulins. All sections were examined under light microscopy (Olympus BX45, Olympus, Hamburg, Germany).

### Biochemical analyses

The aspirates were resuspended in a total volume of 100 μl of fresh DMEM and digested with papain (final concentration 75 μg/ml) at 60 °C (Frisch et al. 2015b). The DNA contents were measured by fluorimetry using Hoechst 22358 and the proteoglycan contents by binding to dimethylmethylene blue dye (Frisch et al. 2015b). The type-II, −I, and -X collagen contents were determined by ELISA (Frisch et al. 2015a). Values were normalized to total cellular proteins monitored via Pierce Thermo Scientific Protein Assay (Fisher Scientific, Schwerte, Germany). All measurements were performed on a GENios spectrophotometer/fluorometer (Tecan).

### Histological and immunohistochemical analyses

Aspirates were collected and fixed in 4 % formalin with subsequent dehydration in graded alcohols, paraffin embedding and sectioning at 3 μm. Hematoxylin eosin (H&E) staining was performed to evaluate cellularity and toluidine blue and alizarin red staining for the detection of matrix proteoglycans and matrix mineralization, respectively (Frisch et al. 2015a; Rey-Rico et al. 2015). The expression of type-II, −I, and -X collagen and of SOX9 was evaluated by immunohistochemistry using specific primary antibodies, biotinylated secondary antibodies and the ABC method (Frisch et al. 2015b; Rey-Rico et al. 2015). Control conditions were included by omitting the primary antibodies. All sections were examined under light microscopy (Olympus BX45).

### Histomorphometry

Cell proliferation was evaluated by counting the total cells per standardized area on H&E-stained sections (Frisch et al. 2015b). The intensities of H&E, toluidine blue, and alizarin red staining and those of FGF-2, type-II, −I, and -X collagen, and SOX9 immunostaining were monitored at magnification ×20 by inverting the pictures to grayscale mode, adapting background DAB signal for comparable range, and measuring the mean gray value per total area covered with cells (mm^2^) (Frisch et al. 2015b; Rey-Rico et al. 2015). The data were recorded at three random standardized sites or with 10 serial histological and immunohistochemical sections for each parameter, test and replicate condition using the SIS analySIS program (Olympus) and Adobe Photoshop (Adobe Systems, Unterschleissheim, Germany) and are given as mean intensity of staining or immunostaining in pixels per mm^2^ of total cell area (Frisch et al. 2015b; Rey-Rico et al. 2015).

### Real-time RT-PCR analyses

TRIzol reagent (Ambion® Life Technologies) and RNeasy Protect Mini Kit (Qiagen, Hilden, Germany) were used to extract total cellular RNA from all chondrogenically differentiated aspirates on day 21 post-transduction. The procedure included an on-column RNase-free DNase treatment (Qiagen, Hilden, Germany) and extracted RNA was eluted in 30 μl of RNase-free water followed by reverse transcription using the 1st Strand cDNA Synthesis kit for RT-PCR (AMV) (Roche Applied Science) with aliquots of 8 μl RNA eluate. The resulting cDNA products (≥2 μl per sample) were finally amplified by real-time RT-PCR with Brilliant SYBR Green QPCR Master Mix (Stratagene, Agilent Technologies, Waldbronn, Germany) on an Mx3000P QPCR operator system (Stratagene) under the following conditions: (95 °C, 10 min), amplification by 55 cycles (denaturation at 95 °C, 30 s; annealing at 55 °C, 1 min; extension at 72 °C, 30 s), denaturation (95 °C, 1 min), and final incubation (55 °C, 30 s). Primers for selected gene profiles are listed in Table 1 and applied at a final concentration of 150 nm. Controls consisting of water and non-reverse-transcribed mRNA were included and confirmation of the product specificities was done via melting curve analysis and agarose gel electrophoresis as previously described (Frisch et al. 2015b). The MxPro QPCR Software (Stratagene) was used for measurements of the threshold cycle (Ct) value of each gene of interest and all values were normalized to GAPDH expression using the 2^-ΔΔCt^ method (Frisch et al. 2015b).

**Table 1:** Primers used for real-time RT-PCR

Gene	Primer sequences (5’-3’)
ACAN^a^	GAGATGGAGGGTGAGGTC ACGCTGCCTCGGGCTTC
COL2A1^a^	GGACTTTTCTCCCCTCTCT GACCCGAAGGTCTTACAGGA
SOX9^a^	ACACACAGCTCACTCGACCTTG GGGAATTCTGGTTGGTCCTCT
COL1A1^b^	ACGTCCTGGTGAAGTTGGTC ACCAGGGAAGCCTCTCTCTC
COL10A1^c^	CCCTCTTGTTAGTGCCAACC AGATTCCAGTCCTTGGGTCA
MMP13^c^	AATTTTCACTTTTGGCAATGA CAAATAATTTATGAAAAAGGGATGC
ALP^b^	TGGAGCTTCAGAAGCTCAACACCA ATCTCGTTGTCTGAGTACCAGTCC
RUNX2^b^	GCAGTTCCCAAGCATTTCAT CACTCTGGCTTTGGGAAGAG
GAPDH^d^	GAAGGTGAAGGTCGGAGTC GAAGATGGTGATGGGATTTC

*SOX9* SRY (sex determining region Y)-box 9, *COL2A1* type-II collagen α1, *ACAN* aggrecan, *COL1A1* type-I collagen α1, *COL10A1* type-X collagen α1, *MMP13* matrix metallo-proteinase 13, *ALP* alkaline phosphatase, *RUNX2* runt-related transcription factor 2, *GAPDH* glyceraldehyde-3-phosphate dehydrogenase. ^a^Chondrogenic markers; ^b^Osteogenic markers; ^c^Hypertrophic and terminal differentiation markers; ^d^Housekeeping gene (control)

### Statistical analysis

Each condition was performed in duplicate in two independent experiments for each patient. Data are expressed as mean ± standard deviation (SD) of separate experiments. The *t*-test and Mann–Whitney Rank Sum Test were used where appropriate. Any *P* value of less than 0.05 was considered statistically significant.

## Results

### Effective genetic modification of chondrogenically-induced human bone marrow aspirates to overexpress FGF-2 via rAAV gene transfer

**Fig. 1:**
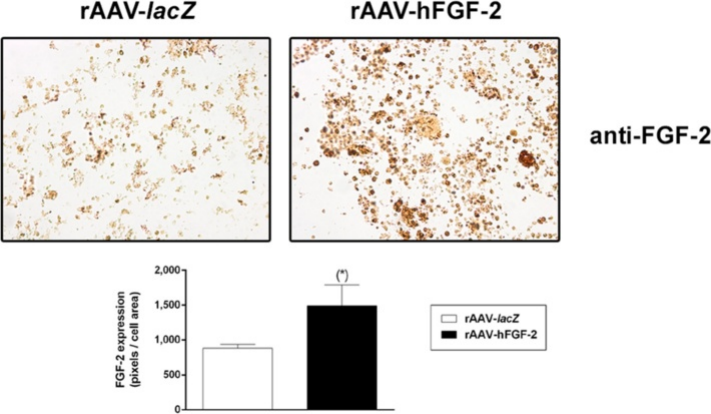
Evaluation of FGF-2 overexpression in rAAV-transduced, chondrogenically-induced human bone marrow aspirates. The aspirates were transduced with rAAV-*lacZ* or rAAV-hFGF-2 (40 μl each vector) and kept in chondrogenic medium for 21 days. The samples were processed to monitor FGF-2 production by immunohistochemical analysis (magnification ×20; representative data) with corresponding histomorphometric assessments as described in the Methods. *Statistically significant compared with rAAV-*lacZ*

Human bone marrow aspirates were first transduced with the candidate rAAV-hFGF-2 vector versus control (rAAV-*lacZ*) condition and induced towards chondrogenesis for up to 21 days to test the ability of rAAV to mediate the overexpression of the candidate FGF-2. An analysis of transgene expression revealed significantly higher immunoreactivity to FGF-2 in the aspirates transduced with rAAV-hFGF-2 compared with rAAV-*lacZ* (1.7-fold difference, *P* = 0.009) (Fig. 1). These findings were corroborated by measurements of the FGF-2 production levels, with significantly higher values upon FGF-2 gene transfer versus *lacZ* at any time point of the analysis (up to 4.1-fold difference, *P* ≤ 0.003) (Table 2).

**Table 2:** Analysis of the levels of FGF-2 production in rAAV-transduced, chondrogenically-induced human bone marrow aspirates

Days post-transduction	rAAV-*lacZ*	rAAV-hFGF-2
7	23.6 (4.5)	96.7 (10.3)^a^
14	20.0 (1.5)	31.2 (2.4)^a^
21	22.2 (2.5)	36.2 (7.8)^a^

Values are expressed as means pg/mg total proteins/24 h (SD). ^a^Statistically significant compared with rAAV-*lacZ*

### Effects of FGF-2 overexpression via rAAV upon the proliferation and chondrogenic differentiation of human bone marrow aspirates

Chondrogenically-induced aspirates were next transduced with rAAV-hFGF-2 relative to rAAV-*lacZ* in order to monitor the proliferative activities and differentiation events in the samples following FGF-2 overexpression. As we previously reported a lack of deleterious effects of rAAV gene transfer upon the potency of bone marrow aspirates (Rey-Rico et al. 2015), we did not test a condition lacking vector treatment in this study.

**Fig. 2:**
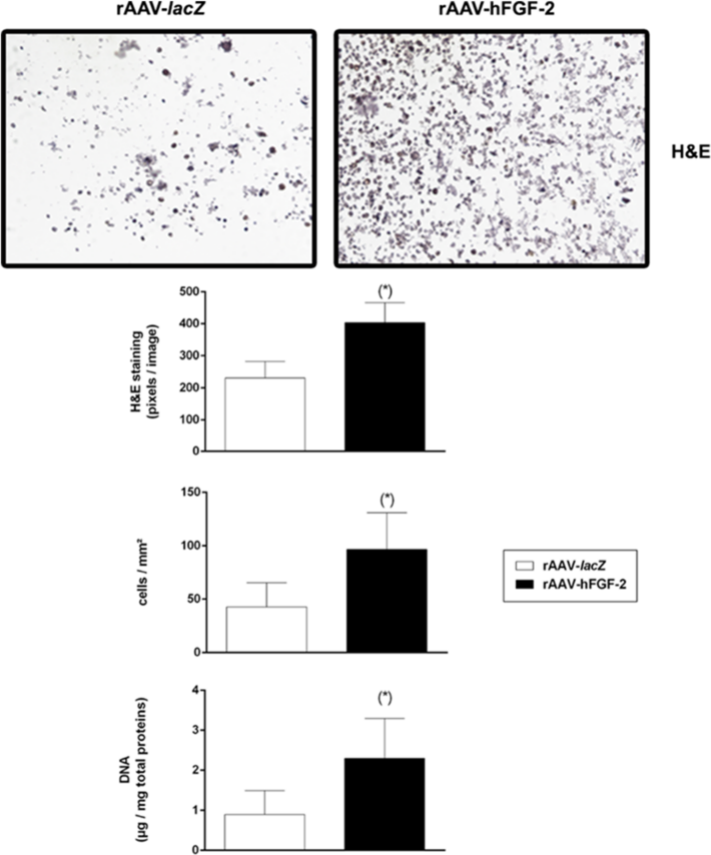
Effects of FGF-2 overexpression upon the proliferative activities in rAAV-transduced, chondrogenically-induced human bone marrow aspirates. The aspirates were transduced with rAAV-*lacZ* or rAAV-hFGF-2 as described in Fig. 1 and kept in chondrogenic medium for 21 days. The samples were processed to evaluate the cell densities on H&E-stained histological sections (magnification ×20; representative data) with corresponding histomorphometric assessments and to monitor the DNA contents as described in the Methods. *Statistically significant compared with rAAV-*lacZ*

Quantitative assessment of the intensities of H&E staining and of the cell densities on histological sections from aspirates demonstrated significantly higher values with rAAV-hFGF-2 compared with rAAV-*lacZ* (up to 2.3-fold difference, *P* ≤ 0.027) (Fig. 2). These results were supported by an estimation of the DNA contents in the aspirates with significantly higher values upon overexpression of FGF-2 (2.6-fold difference, *P* = 0.046) (Fig. 2).

**Fig. 3:**
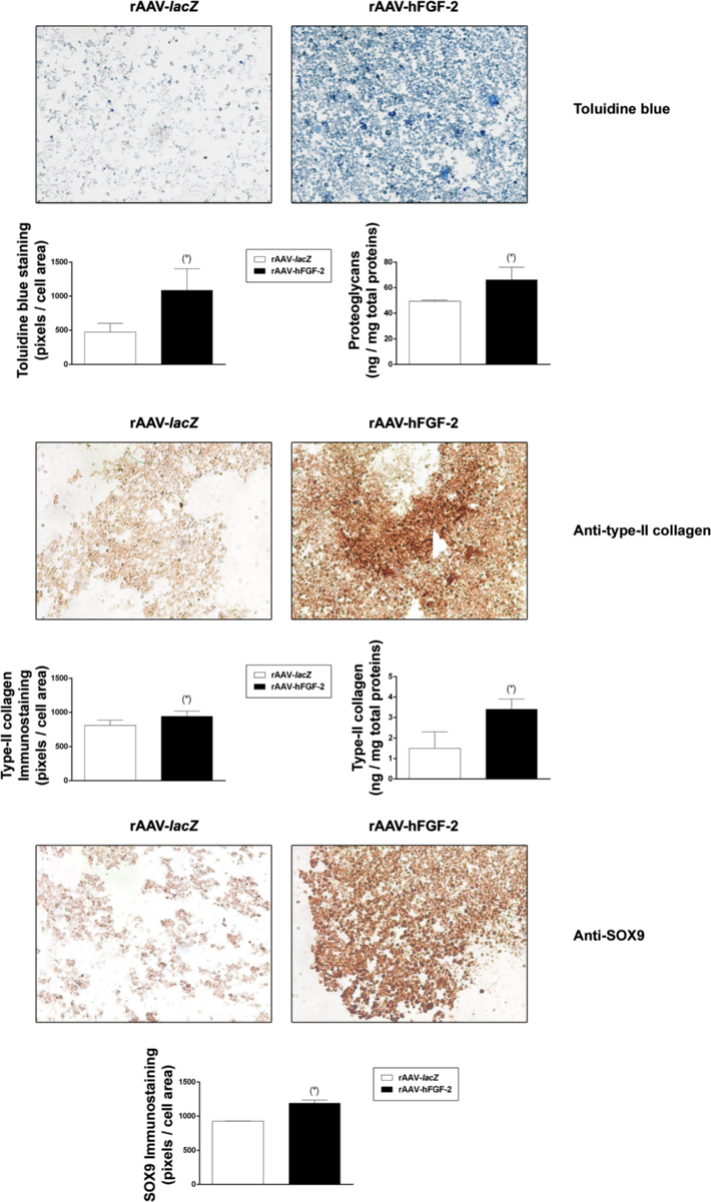
Effects of FGF-2 overexpression upon the production of cartilage-specific components in rAAV-transduced, chondrogenically-induced human bone marrow aspirates. The aspirates were transduced with rAAV-*lacZ* or rAAV-hFGF-2 as described in the Figs. 1 and 2 and kept in chondrogenic medium for 21 days. The samples were processed to monitor the deposition of proteoglycans and of type-II collagen (toluidine blue staining and immunodetection, respectively) and the expression of SOX9 (immunodetection) (all at magnification ×20; representative data) with corresponding histomorphometric assessments and to estimate the proteoglycan and type-II collagen contents as described in the Methods. *Statistically significant compared with rAAV-*lacZ*

To examine the influence of FGF-2 overexpression on the chondrogenic events in the aspirates, the samples were processed to determine the levels of proteoglycan and type-II collagen deposition and the levels of SOX9 expression. Significantly increased intensities were noted in the presence of rAAV-hFGF-2 relative to rAAV-*lacZ* for toluidine blue staining (2.3-fold difference, *P* = 0.016), type-II collagen immunostaining (1.2-fold difference, *P* = 0.002), and SOX9 immunostaining (1.3-fold difference, *P* = 0.037) (Fig. 3). These findings were substantiated by an estimation of the proteoglycan and type-II collagen contents (up to 2.3-fold difference, *P* ≤ 0.022) (Fig. 3). Finally, real-time RT-PCR analyses revealed 3.6-, 4.9-, and 1.9-fold increases in the gene expression profiles of ACAN, COL2A1, and SOX9, respectively (*P* ≤ 0.018) when applying rAAV-hFGF-2 compared with rAAV-*lacZ* (please see Fig. 5).

### Effects of FGF-2 overexpression via rAAV upon the hypertrophic and terminal differentiation of human bone marrow aspirates

Chondrogenically-induced aspirates treated with rAAV-hFGF-2 versus rAAV-*lacZ* were finally processed to evidence possible effects of transgenic FGF-2 expression upon possible hypertrophic and terminal differentiation events in the samples.

**Fig. 4:**
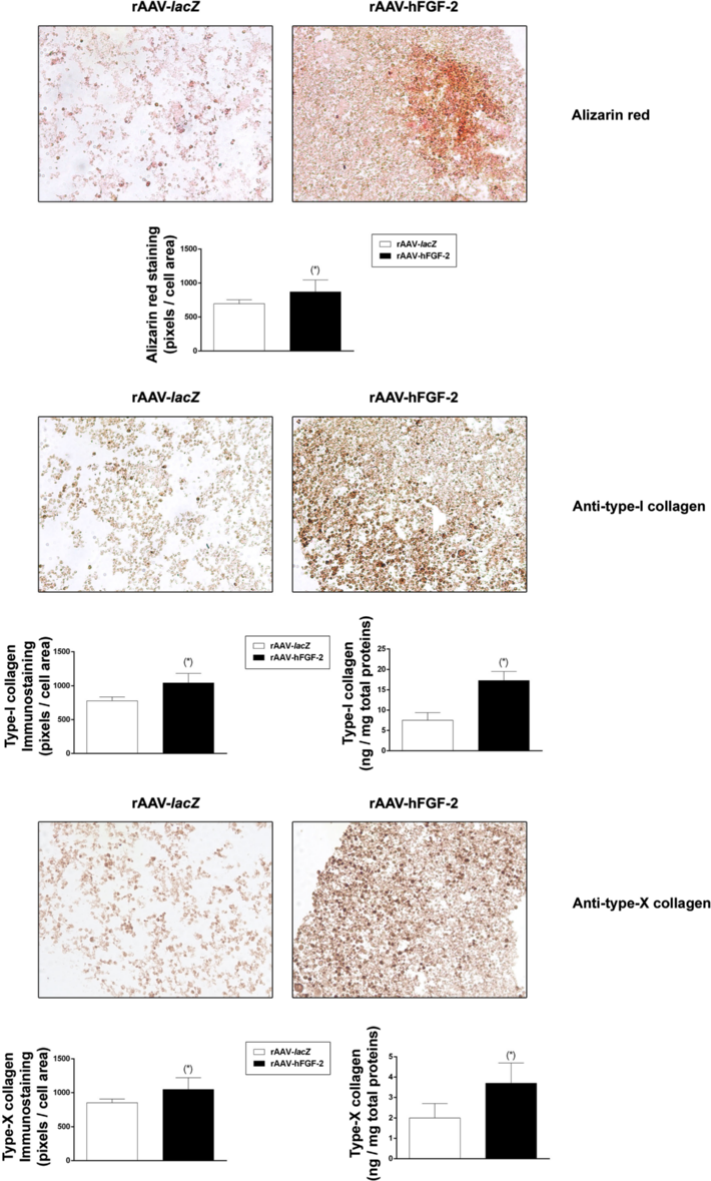
Effects of FGF-2 overexpression upon the hypertrophic and terminal differentiation processes in rAAV-transduced, chondrogenically-induced human bone marrow aspirates. The aspirates were transduced with rAAV-*lacZ* or rAAV-hFGF-2 as described in the Figs. 1, 2, and 3 and kept in chondrogenic medium for 21 days. The samples were processed to monitor matrix mineralization (alizarin red staining) and the expression of type-I and type-X collagen (immunodetection) (all at magnification ×20; representative data) with corresponding histomorphometric assessments and to estimate the type-I and -X collagen contents as described in the Methods. *Statistically significant compared with rAAV-*lacZ*

**Fig. 5:**
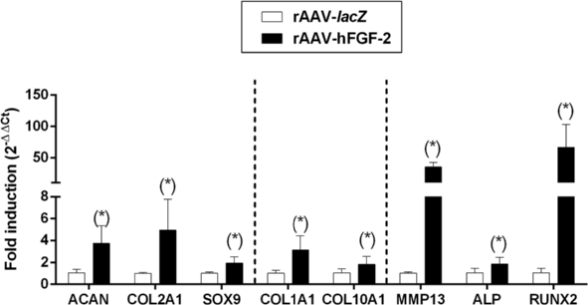
Gene expression analyses by real-time RT-PCR in rAAV-transduced, chondrogenically-induced human bone marrow aspirates overexpressing FGF-2. The aspirates were transduced with rAAV-*lacZ* or rAAV-hFGF-2 as described in the Figs. 1, 2, 3, and 4 and kept in chondrogenic medium for 21 days. The samples were processed to monitor the expression profiles of aggrecan (ACAN), type-II collagen (COL2A1), the transcription factor SOX9, type-I collagen (COL1A1), type-X collagen (COL10A1), matrix metalloproteinase 13 (MMP13), alkaline phosphatase (ALP), and the transcription factor RUNX2, with GAPDH serving as a housekeeping gene and internal control for normalization. Ct values were generated for each target gene and fold inductions (relative to rAAV-*lacZ*-treated aspirates) were measured by using the 2^-ΔΔCt^ method as described in the Methods. *Statistically significant compared with rAAV-*lacZ*

Significantly higher intensities were seen with rAAV-hFGF-2 compared with rAAV-*lacZ* for alizarin red staining (1.2-fold difference, *P* = 0.017), type-I collagen immunostaining (1.3-fold difference, *P* = 0.006), and type-X collagen immunostaining (1.2-fold difference, *P* = 0.040) (Fig. 4). These findings were corroborated by an estimation of the type-I and -X collagen contents (up to 2.3-fold difference, *P* ≤ 0.028) (Fig. 3). Real-time RT-PCR analyses also showed 3.1- and 1.7-fold increases in the gene expression profiles of COL1A1 and COL10A1, respectively (*P* ≤ 0.040) with rAAV-hFGF-2 relative to rAAV-*lacZ* (Fig. 5). Such enhanced profiles with FGF-2 were associated with higher levels of MMP13, ALP, and RUNX2 expression as markers of terminal differentiation and osteogenesis (34.9-, 1.8-, and 62.5-fold, respectively, *P* ≤ 0.023) (Fig. 5).

## Discussion

Therapeutic gene transfer via clinically adapted rAAV vectors is an attractive strategy to effectively and durably enhance the chondrogenic processes in bone marrow concentrates prior to re-implantation in sites of cartilage injury (Cucchiarini et al. 2014; Frisch et al. 2015a). The goal of this study was to demonstrate the possibility of applying a functional rAAV construct carrying the sequence for the proliferative and pro-chondrogenic FGF-2 (Solchaga et al. 2010; Solchaga et al. 2005; Tsutsumi et al. 2001) to freshly prepared human marrow aspirates in light of our previous findings in isolated human MSCs (Cucchiarini et al. 2011) and of evidence showing the potential of using such an approach to deliver reporter genes and other candidate sequences (SOX9, IGF-I) (Frisch et al. 2015b; Rey-Rico et al. 2015).

The results demonstrate that significant levels of FGF-2 expression were achieved in the aspirates upon rAAV-mediated gene transfer compared with control (*lacZ*) transduction over the whole period of evaluation (21 days), probably due to a good maintenance of this vector class in these targets (Rey-Rico et al. 2015), in agreement with our previous findings using the same vector to target isolated human MSCs (Cucchiarini et al. 2011). The decrease in FGF-2 expression noted over time may be due to a dilution of the transgene sequences upon cell proliferation and expansion as the rAAV vectors are mostly kept under (stable) episomal forms in their targets. Interestingly, the amounts of factor produced in the aspirates over time following rAAV-hFGF-2 application (~36 pg rhFGF-2/mg total proteins/24 h after 21 days in chondrogenic medium) were lower than those reported in MSCs (~137 pg/ml/24 h using 2 × 10^5^ cells in chondrogenically-induced aggregate cultures, i.e. ~ 360 pg rhFGF-2/mg total proteins/24 h and a 10-fold difference) (Cucchiarini et al. 2011), reflecting the presence of other cell subpopulations in the aspirates (hematopoietic progenitor cells, hematopoietic cells, fibroblasts) that may not be a source of FGF-2 expression after 21 days of continuous chondrogenic induction as mostly the MSCs specifically and extensively commit toward the chondrocyte phenotype in this environment (Anam and Davis 2013; Frisch et al. 2015b; Lennon et al. 2000), being the major contributor of production over time.

Such sustained levels of FGF-2 generated from rAAV gene delivery were capable of stimulating the proliferative, biosynthetic, and chondrogenic activities in the aspirates over time versus *lacZ* treatment, consistent with the effects of FGF-2 when used in a recombinant form (Solchaga et al. 2010; Solchaga et al. 2005; Tsutsumi et al. 2001) or following application of the candidate rAAV-hFGF-2 construct to isolated human MSCs (Cucchiarini et al. 2011). The levels of proliferation reached over time in the aspirates via FGF-2 gene transfer were higher than those observed in isolated MSCs (~2.3 μg versus ~ 1.1 ng DNA/mg total proteins after 21 days, respectively, i.e. an ~ 2.1 × 10^3^-fold difference) (Cucchiarini et al. 2011), probably due to the presence of other mitogenic factors in the samples secreted by all marrow populations. Also, the levels of matrix biosynthesis in the aspirates mediated over time by FGF-2 overexpression were higher than those noted in isolated MSCs (~1087 versus ~ 170 pixels of standardized toluidine blue staining, ~ 66 versus ~ 0.9 ng proteoglycans/mg total proteins, and ~ 3.4 versus ~ 0.025 ng type-II collagen/mg total proteins on day 21, respectively, i.e. an up to 136-fold difference) (Cucchiarini et al. 2011), again possibly due to a more favorable biochemical and cellular environment for MSC chondrogenesis in heterogeneous aspirates or to an up-regulation of the levels of highly chondrogenic transcription factor SOX9 (Bell et al. 1997; Lefebvre et al. 1997) by FGF-2 in these samples versus isolated MSCs (Cucchiarini et al. 2011), concordant with the reported stimulating effects of the growth factor on the activity of the *sox9* gene (Murakami et al. 2000).

Interestingly, the results also indicate that administration of the rAAV FGF-2 construct promoted the development of hypertrophic and terminal differentiation events in the aspirates in the particular conditions selected, with enhanced levels of MMP13, ALP, and RUNX2 (markers of terminal and osteogenic differentiation, transcription factor controlling the osteoblastic expression of COL1, COL10, and MMP13) as previously described with recombinant FGF-2 (Solchaga et al. 2010; Solchaga et al. 2005; Tsutsumi et al. 2001) but in contrast with our findings when applying the current vector to isolated MSCs (Cucchiarini et al. 2011). Such discrepancy may be due to pro-hypertrophic activities induced by other marrow subpopulations.

This study demonstrate the possibility of delivering a candidate FGF-2 sequence to human bone marrow concentrates via rAAV vectors as a means to expand the processes of chondrogenic differentiation in such samples in vitro. It remains to be seen whether prolonged FGF-2 secretion via rAAV may not lead to side (paracrine and autocrine) effects (activation of proliferation/differentiation of undesirable cell populations), even though we did not observe such problems when applying directly the current vector to focal experimental articular cartilage defects in vivo (Cucchiarini et al. 2005). Work is now ongoing to define the subpopulations participating in the chondrogenic events in order to investigate cell heterogeneity within the aspirates (Anam and Davis 2013; Jones et al. 2002; Lennon et al. 2000). We are also currently testing the feasibility of translating the approach in vivo by implanting genetically modified marrow aspirates within experimental models of articular cartilage defects to corroborate the observations in vitro (Cucchiarini et al. 2013; Ivkovic et al. 2010; Pascher et al. 2004; Sieker et al. 2015). As application of rAAV FGF-2 led to premature hypertrophy and terminal differentiation in the aspirates, controlled expression of this factor as afforded by using tissue-specific (type-II collagen, SOX9) or regulatable promoters (tetracycline-sensitive) may allow to restrain such possible, undesirable events in vivo. Alternatively, combined gene transfer using extra, anti-hypertrophic agents might be capable of circumventing these effects in vivo like by co-applying the SOX9 transcription factor (Cucchiarini et al. 2013; Rey-Rico et al. 2015). Overall, the present findings provide evidence on the benefits of conveniently modifiying marrow aspirates by rAAV gene transfer for future applications to treat articular cartilage defects.

## Conclusions

Genetic modification of bone marrow aspirates is a promising avenue of translational research to treat cartilage defects in patients especially using clinically adapted therapeutic rAAV vectors. The current study provides evidence of the possibility to deliver the proliferative and pro-chondrogenic FGF-2 to primary human marrow aspirates via rAAV for the durable commitment of the samples toward chondrogenic differentiation. The occurrence of hypertrophic events in vitro suggests that controlled expression of the growth factor will be a prerequisite for future applications in vivo to effectively treat cartilage lesions in the affected human population.
